# Identification of novel clinical subtypes in patients with microscopic polyangiitis using cluster analysis: multicenter REVEAL cohort study

**DOI:** 10.3389/fimmu.2024.1450153

**Published:** 2025-01-20

**Authors:** Ayana Okazaki, Shogo Matsuda, Takuya Kotani, Keisuke Fukui, Takaho Gon, Ryu Watanabe, Atsushi Manabe, Mikihito Shoji, Keiichiro Kadoba, Ryosuke Hiwa, Wataru Yamamoto, Motomu Hashimoto, Tohru Takeuchi

**Affiliations:** ^1^ Department of Internal Medicine IV, Division of Rheumatology, Osaka Medical and Pharmaceutical University, Takatsuki, Japan; ^2^ Faculty of Societal Safety Sciences, Kansai University, Takatsuki, Japan; ^3^ Department of Medical Statistics, Research & Development Center, Osaka Medical and Pharmaceutical University, Takatsuki, Japan; ^4^ Department of Clinical Immunology, Osaka Metropolitan University Graduate School of Medicine, Osaka, Japan; ^5^ Department of Rheumatology and Clinical Immunology, Kyoto University Graduate School of Medicine, Kyoto, Japan; ^6^ Department of Health Information Management, Kurashiki Sweet Hospital, Kurashiki, Japan

**Keywords:** the running head: clinical subtypes in MPA (microscopic polyangiitis), principal component analysis, cluster analysis, prognosis, real world evidence

## Abstract

**Introduction:**

This study aimed to identify new clinical phenotypes of microscopic polyangiitis (MPA) using a principal components analysis (PCA)-based cluster analysis.

**Methods:**

A total of 189 patients with MPA between May 2005 and December 2021 were enrolled from a multicenter cohort in Japan (REVEAL cohort). Categorical PCA and cluster analysis were performed based on clinical, laboratory, and radiological findings. Clinical characteristics and outcomes, including all-cause mortality, respiratory-related mortality, end-stage renal disease (ESRD), and relapse were compared between each cluster.

**Results:**

Eleven clinical variables were transformed into four components using categorical PCA and synthetic variables were created. Additionally, a cluster analysis was performed using these variables to classify patients with MPA into subgroups. Four distinct clinical subgroups were identified: Cluster 1 included the renal involvements and diffuse alveolar hemorrhage (DAH)-dominant group (N=33). Cluster 2 comprised the elderly onset systemic inflammation group (N=75). Cluster 3 included patients in the younger-onset limited-organ disease group (N=45). Cluster 4 was comprised of an ILD-predominant group without kidney involvement (N=36). 61 patients died during follow-up, with 32 dying of respiratory-related causes. Additionally, 19 patients developed ESRD and 70 relapsed. Cluster 1 showed the worst ESRD-free survival and relapse rates, whereas Cluster 2 showed the worst overall survival and respiratory-related death-free survival rates among the four groups.

**Conclusions:**

Our study identified four unique subgroups with different MPA outcomes. Individualized treatments for each subgroup may be required to improve the prognosis of MPA.

## Introduction

Microscopic polyangiitis (MPA) is a subtype of anti-neutrophil cytoplasmic antibody (ANCA)-associated vasculitis (AAV) that predominantly affects small vessels with few or no immune deposits and involves several organs, such as the skin, lungs, and kidneys (1). The major target antigens of ANCA in AAV are myeloperoxidase (MPO) and proteinase 3 (PR3). MPA is commonly associated with MPO-ANCA (2). In Asian countries, the proportion of MPO-ANCA positive MPA is higher in patients with AAV (3). Also, kidney and pulmonary involvements are the most common manifestations of MPO-ANCA-positive MPA (1, 4).

MPA is a heterogeneous disease because its clinical presentation ranges from limited to generalized phenotypes. Prognosis varies according to organ involvement in MPA (5). MPA patients with localized involvement, such as ear, nose, and throat (ENT) lesions, respond to immunosuppressive therapy (6). In contrast, rapidly progressive glomerulonephritis (RPGN) and diffuse alveolar hemorrhage (DAH) are poor prognostic factors in patients with MPA and require high-dose immunosuppressive therapy and plasma exchange (7, 8). EUVAS (European Vasculitis Study Group) has previously defined clinical subgroups based on systemic involvement in clinical trials of GPA (Granulomatosis with polyangiitis), but it has not been elucidated whether differences in clinical background determine prognosis in MPA (9). Therefore, the stratification of patients based on their clinical background is crucial for improving the prognosis of MPA.

Cluster analysis is a method frequently used for grouping patients into homogeneous subgroups. Previous studies have shown that cluster analysis divides patients with AAV into subgroups based on clinical characteristics and that they have different prognoses (10, 11). However, these data include patients with AAV, including those with GPA and MPA; therefore, there are few reports which focus on subcategorizing patients with heterogeneous MPA based on clinical characteristics.

In the present study, Principal Component Analysis (PCA)-based cluster analysis was conducted to identify new clinical subgroups based on clinical characteristics in MPA using the multicenter database. Herein, four new subgroups with different clinical phenotypes and prognoses were identified.

## Materials and methods

### Patients

This multicenter, observational, and retrospective study was conducted in the Registry of Vasculitis Patients to Establish REAL World Evidence (REVEAL) cohort to identify subgroups of patients with MPA based on clinical characteristics. The REVEAL cohort is an observational multicenter registry of patients with MPA in the Kansai District of Japan (12). Data of patients from three participating institutes (Osaka Medical and Pharmaceutical University, Kyoto University, and Osaka Metropolitan University) were included, and 211 patients with MPA were registered between May 2005 and December 2021. The Chapel Hill Consensus definition was used to diagnose MPA (13). Patients with other diagnoses, such as malignant tumors, infectious diseases, drug-induced vasculitis, secondary vasculitis, pseudo-vasculitis, and/or sarcoidosis, were excluded (14). At enrolment, data were retrospectively collected from an electronic database by a reference clinician at each center. All patients were hospitalized for remission induction therapy, and all except one received immunosuppressive treatment at the physician’s discretion. All clinical and laboratory findings, treatments, and outcomes were retrospectively extracted from medical records.

This study was conducted in accordance with the Declaration of Helsinki and its amendments and was approved by the Medicine Ethics Committee of Osaka Medical and Pharmaceutical University (approval no. 1529) and by the individual participating centers, including Kyoto University (approval no. R1540) and Osaka Metropolitan University (approval no. 2023-027). The Ethics Committee of Kyoto University waived the requirement for informed consent because of the anonymous nature of the data. Written informed consent was obtained from each patient at the other institutions.

### Clinical findings and laboratory parameters on admission

In the REVEAL cohort database, the following data were collected from the medical records on the first remission induction therapy: patient demographic characteristics, including age on admission and sex; peripheral laboratory data, including white blood cell (WBC) counts, hemoglobin (Hb), albumin, creatinine, C-reactive protein (CRP), Krebs von den Lungen-6 (KL-6), MPO-ANCA, and proteinase 3 (PR3)-ANCA; systemic organ involvement defined by the Birmingham Vasculitis Activity Score (BVAS), version 3 (15), and the contents of treatments.

### Evaluation of disease severity

We assessed systemic disease activity using BVAS (15). We also evaluated disease severity according to the European Vasculitis Study Group (EUVAS) categorization system (9). Additionally, we also evaluated the 2009 five-factor score (FFS) for each patient, which was used to evaluate prognosis at the time of MPA diagnosis (16).

### Outcome

To evaluate outcomes, follow-up survival data were collected retrospectively from the REVEAL cohort. the overall mortality, respiratory-related mortality, end-stage renal disease (ESRD)-free survival, and relapse-free survival rates were measured. Respiratory-related mortality was defined as described previously (17). ESRD was defined as an estimated glomerular filtration rate (eGFR) <15 mL/min/1.73 m2 and is a requirement for permanent renal replacement therapy. Patients who were dependent on hemodialysis from the time of MPA diagnosis for more than three months were considered to have ESRD, as previously described (18). Relapse was recurrence of vasculitis requiring treatment change, increasing dose of glucocorticoids, and/or adding immunosuppressants due to the exacerbation of symptoms and clinical data (19).

### Variable selection

A previous Japanese multicenter study revealed that MPA was frequently accompanied by systemic symptoms, excluding cardiovascular and abdominal symptoms (20). In addition, MPA is an elderly onset-disease and is often accompanied by interstitial lung disease (ILD) and RPGN (21, 22). Based on these findings, eleven clinical variables were selected for this study, including age, serum CRP levels, serum creatinine levels, presence of ILD, and seven items of the BVAS 2003 (general, cutaneous, mucous membranes/eyes, ENT, chest, renal, and nervous symptoms).

### Categorical PCA

To analyze the relationships between clinical characteristics, categorical principal components analysis (PCA) was used to statistically aggregate items, thus reducing the number of observed continuous and binary variables to a smaller number of principal components (PCs) and reducing the dimensionality of clinical characteristics (23, 24). According to the Kaiser-Guttman rule and the scree plot method, four eigen vectors with eigenvalues greater than 1.0 were selected (25). The eigenvalues of PC1, PC2, PC3, and PC4 were 1.8, 1.6, 1.4, and 1.1, respectively (**Supplementary Figure 1**), and were used to create synthetic variables and were retained for further cluster analysis.

### Cluster analysis

Cluster analyses were performed using the Ward method to identify subgroups of patients with MPA (26). The clustering algorithm begins with each patient as a single cluster. The nearest clusters were merged to form a new cluster and repeated until all data were contained in one cluster. The number of clusters was defined based on a scree plot (**Supplementary Figure 2**). There was a natural break where the distance jumps suddenly, and this point was determined as the cutoff point. The appropriate number of clusters was defined as four. The data were analyzed using JMP 15 software (SAS Institute Inc., Cary, NC, USA).

### Statistical analysis

The data were presented as medians and interquartile ranges. Chi-squared test was used for nominal data, and the Kruskal Wallis test was used to compare median values among four groups. *P* values of <0.05 were considered statistically significant. The Kaplan-Meier method was used to assess survival curves, and the log-rank test was used to evaluate the significance of differences between the two groups. Survival time was calculated from the date of remission induction therapy at each institution and ended at the latest hospital visit, date of censoring, or time of overall mortality, respiratory-related death, ESRD, or relapse. When we compared four clusters, statistical significance was determined by <0.0083 using Bonferroni correction to adjust for multiple testing (22). Data were analyzed using JMP (version 15.0; SAS Institute Inc., Cary, NC, USA) and GraphPad Prism (version 8.0; GraphPad Software, La Jolla, CA, USA). R language Ver. 4.0.3, and the Gifi package was used for the application of categorical PCA (27, 28).

## Results

### Clinical characteristic

In the REVEAL cohort, 211 patients were diagnosed with MPA. Of the 211 cases, 22 were excluded because 21 of them were already receiving prednisolone and immunosuppressant therapy, and one patient had not received immunosuppressant therapy after the diagnosis of MPA. The clinical characteristics of the 189 patients with MPA are shown in **Table 1**. MPO-ANCA was positive in 187 patients, and PR3-ANCA was positive in nine patients. Eight patients were double positive for MPO/PR3-ANCA. The median age of the patients was 73 years and 54.5% were women. The median initial WBC count, serum albumin, serum creatinine, CRP levels, and MPO-ANCA titer were 10,540/mm3, 2.6 g/dL, 1.1 mg/dL, 7.6 mg/dL, 123.0 U/mL, respectively. The median total BVAS score was 14, and the proportions of patients with FFS ≦1, 2, and ≧3 were 19.6%, 55.0%, and 25.4%, respectively. According to the EUVAS-defined disease severity, seven (3.7%), 45 (23.8%), 105 (55.6%), and 32 (16.9%) had localized, early systemic, generalized, and severe disease, respectively. The clinical characteristics between MPA patients diagnosed between 2005 and 2012 (N=51) and those diagnosed between 2013 and 2021(N=138) were shown in **Supplementary Table 1**. The age and the ratio of Five factor score 2009≧3 was significantly higher in patients between 2013 and 2021 compared to those between 2005 and 2012, and there were no differences in the sex ratio, inflammatory markers, organ involvement, and EUVAS-defined disease severity between them. Additional details regarding the treatments are provided in **Supplementary Table 2**.

**Table 1:** Baseline characteristics for 189 patients with MPA in the REVEAL Study.

Characteristics	MPA (n= 189)
Age, years	73 (68-79)
Female, n (%)	103 (54.5)
ILD, n (%)	106 (56.1)
UIP pattern, n (%)	74 (39.2)
Laboratory findings	
WBC,/mm^3^	10,540 (7,900-14,145)
Hb, g/dL	10.1 (8.7-11.8) ^a^
Alb, g/dL	2.6 (2.2-3.2)
Cr, mg/dL	1.1 (0.7-2.1)
CRP, mg/mL	7.6 (3.0-12.3)
Positive anti-MPO-ANCA, n (%)	187 (98.9)
Positive anti-PR3-ANCA, n (%)	9 (4.8)
MPO-ANCA titer, U/mL	123.0 (57.2-250.3) ^b^
Systemic symptoms	
General, n (%)	121 (64.0)
Cutaneous, n (%)	18 (9.5)
Mucous Membranes/eyes, n (%)	13 (6.9)
ENT, n (%)	29 (15.3)
Chest, n (%)	70 (37.0)
Cardiovascular, n (%)	5 (2.6)
Abdominal, n (%)	1 (0.5)
Renal, n (%)	135 (71.4)
Nervous system, n (%)	70 (37.0)
BVAS at onset	14 (8-19)
Five factor score 2009	
≦1	37 (19.6)
2	104 (55.0)
≧3	48 (25.4)
EUVAS-defined disease severity	
Localized	7 (3.7)
Early systemic	45 (23.8)
Systemic	105 (55.6)
Severe	32 (16.9)

The laboratory markers are presented as the median (interquartile range). MPA, microscopic polyangiitis; ILD, interstitial lung disease; WBC, white blood cell; Hb, hemoglobin; Alb, albumin; Cr, creatinine; CRP, C-reactive protein; MPO-ANCA, myeloperoxidase-anti-neutrophil cytoplasmic autoantibody; PR3-ANCA, proteinase 3-anti-neutrophil cytoplasmic antibody; ENT, Ear, Nose and Throat; BVAS, Birmingham Vasculitis Activity Score; EUVAS, European Vasculitis Study Group. ^a^Number of subjects, n= 188. ^b^Number of subjects, n= 186.

### Categorical PCA and synthetic variables

Categorical PCA was conducted using 11 clinical variables, and retained four components with an eigen value >1, consequently explaining 52.38% of the variance in the data. The correlations between the 11 variables and their components are presented in **Supplementary Table 3**. The variables with loadings of at least 0.55 in the absolute value of each component are listed in order in **Supplementary Table 3**. For example, component 1 was mostly correlated with renal symptoms, age, and ILD, and component 2 was mostly correlated with neuronal symptoms and serum CRP levels. Synthetic variables for each patient were created based on the four principal components.

### Cluster analysis of MPA patients

**Figure 1:**
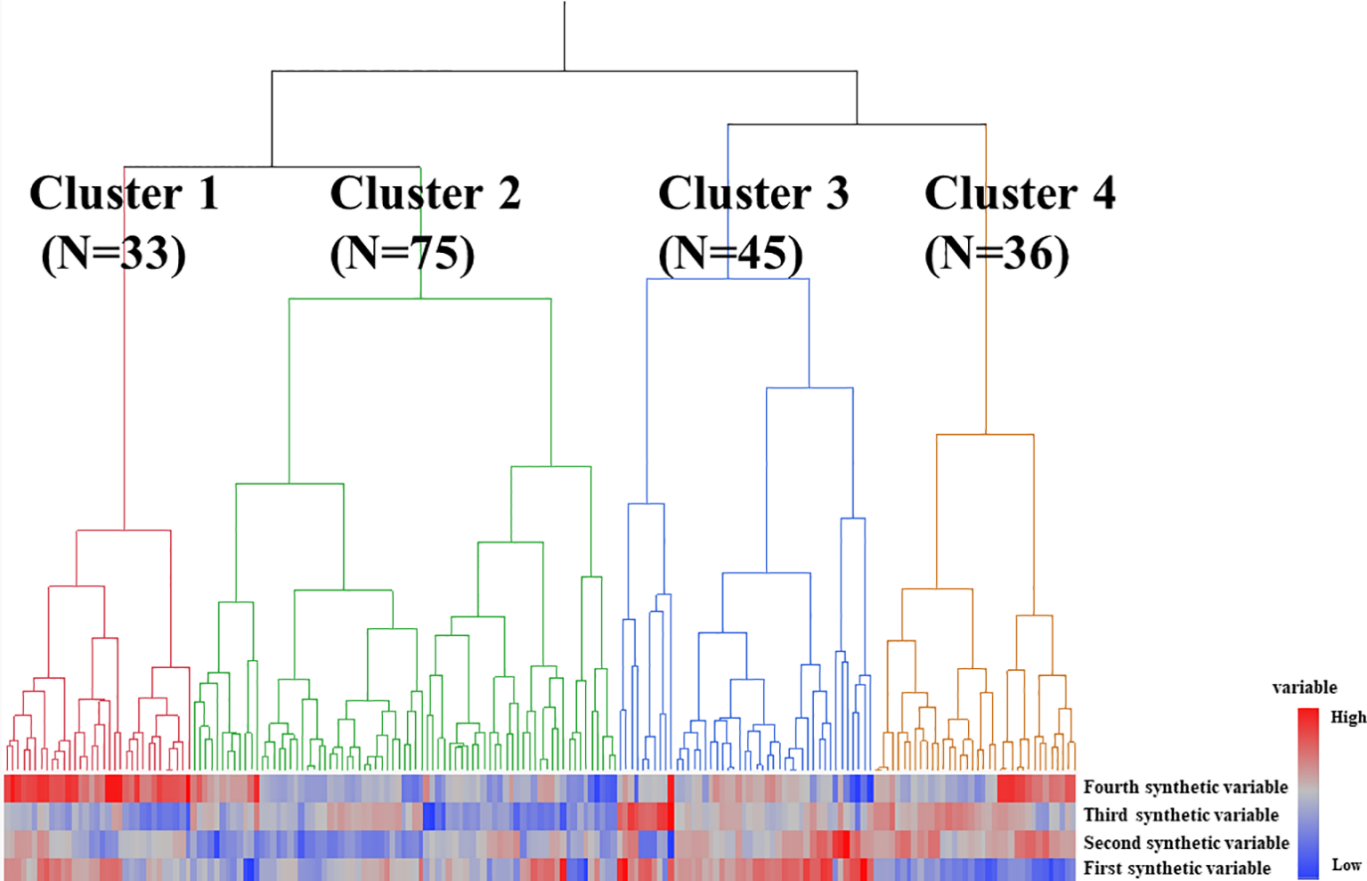
Results of statistical cluster analysis based on synthetic variables in patients with MPA. Groups 1, 2, 3, and 4 are colored red, green, blue, and orange, respectively.

Next, cluster analysis was used to identify subgroups of patients with MPA based on their synthetic variables (**Figure 1**). Individual patients were divided into four groups as shown in **Figure 1**. A comparison of the synthetic variables between Clusters 1, 2, 3, and 4 is presented in **Supplementary Table 4**. The first synthetic variable was highest in Cluster 3, the second and third synthetic variables were highest in Cluster 4, and the fourth was highest in Cluster 1.

### Comparison of the clinical characteristics and prognosis between Clusters 1, 2, 3, and 4 in patients with MPA

The clinical characteristics of patients with MPA in Clusters 1, 2, 3, and 4 were compared (**Table 2**). Cluster 1 (N=33) had the highest prevalence of kidney involvement, highest creatinine levels (median serum Cr levels:1.90 mg/dl), highest prevalence of chest involvement (90.9%), and highest prevalence of DAH (36.4%). Cluster 2 (N=75) was characterized by oldest age (median age:78 years), highest elevation of inflammatory markers (median serum CRP levels:9.5 mg/mL), frequent complications of ILD (61.3%), and kidney involvements accompanied with an elevation of serum creatinine levels (88.0%, 1.26 mg/dl), as well as the highest ENT and neuron symptoms (30.7%, 65.3%, respectively). Cluster 3 (N=45) was characterized by young age and the highest prevalence of cutaneous and mucous membranes/eyes symptoms (22.2% and 28.9%, respectively). Cluster 4 (N=36) was characterized by the highest prevalence of ILD (88.9%) and non-renal complications of kidney involvement. A summary of the clinical characteristics of the four clusters is shown in **Table 3**. Additional details regarding the treatments are provided in **Supplementary Table 5**. Based on these clinical and laboratory findings, four subgroups were identified: Cluster 1, the renal involvement and diffuse alveolar hemorrhage (DAH)-dominant group; Cluster 2, the elderly onset systemic inflammation group; Cluster 3, the younger-onset limited-organ disease group; and Cluster 4, the ILD-predominant group without kidney involvement.

**Table 2:** Comparison of clinical characteristics between cluster 1 and 2 and 3 and 4 cases.

Characteristics	Cluster 1 (n=33)	Cluster 2 (n=75)	Cluster 3 (n=45)	Cluster 4 (n=36)	*P* value
Age, years	72 (67-77)	78 (73-82)	68 (64-72)	74 (66-78)	<0.0001***
Female, n (%)	14 (42.4)	39 (52.0)	29 (64.4)	21 (58.3)	0.25
Laboratory findings					
WBC,/mm^3^	9,500 (7,355-11,750)	12,500 (8,600-16,690)	9,740 (6,885-12,910)	10,540 (7,990-14,265)	0.01*
Alb, g/dl	2.8 (2.5-3.3)	2.4 (1.9-2.9)	2.8 (2.4-3.4)	2.9 (2.4-3.5)	<0.0001***
Cr, mg/dl	1.90 (0.92-5.68)	1.26 (0.79-2.10)	1.16 (0.69-2.42)	0.76 (0.56-0.91)	<0.0001***
CRP, mg/dl	6.3 (2.6-11.2)	9.5 (5.5-13.5)	5.9 (0.5-11.3)	5.8 (0.9-12.0)	0.024*
Complications					
ILD, n (%)	13 (39.4)	46 (61.3)	15 (33.3)	32 (88.9)	<0.0001***
DAH, n (%)	12 (36.4)	7 (9.3)	1 (2.2)	2 (5.6)	<0.0001***
RPGN, n (%)	17 (51.5)	35 (46.7)	15 (33.3)	2 (5.6)	<0.0001***
Systemic symptoms					
General, n (%)	13 (39.4)	55 (73.3)	28 (62.2)	25 (69.4)	0.007**
Cutaneous, n (%)	2 (6.1)	4 (5.3)	10 (22.2)	2 (5.6)	0.011*
Mucous Membranes/eyes, n (%)	0 (0)	0 (0)	13 (28.9)	0 (0)	<0.0001***
ENT, n (%)	1 (3.0)	23 (30.7)	5 (11.1)	0 (0)	<0.0001***
Chest, n (%)	30 (90.9)	19 (25.3)	5 (11.1)	16 (44.4)	<0.0001***
Cardiovascular, n (%)	1 (3.0)	4 (5.3)	0 (0)	0 (0)	0.23
Abdominal, n (%)	0 (0)	1 (1.3)	0 (0)	0 (0)	0.68
Renal, n (%)	33 (100)	66 (88.0)	36 (80.0)	0 (0)	<0.0001***
Nervous system, n (%)	2 (6.1)	49 (65.3)	9 (20.0)	10 (27.8)	<0.0001***
Disease severity					
BVAS	17 (14.5-20)	19 (13-23)	12 (8-14.5)	6 (3-7.8)	<0.0001***
FFS≦1, n (%)	3 (9.1)	12 (16.0)	15 (33.3)	7 (19.4)	0.04*
FFS=2, n (%)	14 (42.4)	43 (57.3)	19 (42.2)	28 (77.8)	0.0051**
FFS≧3, n (%)	16 (48.5)	20 (26.7)	11 (24.4)	1 (2.8)	0.0003***
EUVAS-defined disease severity					
Localized	1 (3.0)	0 (0)	1 (2.2)	5 (13.9)	0.0034**
Early systemic	6 (18.2)	12 (16.0)	14 (31.1)	13 (36.1)	0.06
Systemic	14 (42.4)	51 (68.0)	26 (57.8)	14 (38.9)	0.011*
Severe	12 (36.4)	12 (16.0)	4 (8.9)	4 (11.1)	0.0079**
Prognosis					
Overall mortality	12 (36.4)	34 (45.3)	7 (15.6)	8 (22.2)	0.0036**
Respiratory-related death	4 (12.1)	20 (26.7)	3 (6.7)	5 (13.9)	0.027*
ESRD	7 (21.2)	5 (6.7)	7 (15.6)	0 (0)	0.012*
Relapse	14 (42.4)	30 (40.0)	20 (44.4)	6 (16.7)	0.043*

The laboratory markers are presented as the median (interquartile range). The *P*-values were estimated using Kruskal Wallis test or chi-squared test. *P < 0.05, **P < 0.01, ***P < 0.001. MPA, microscopic polyangiitis; WBC, white blood cell; Alb, albumin; Cr, creatinine; CRP, C-reactive protein; ILD, interstitial lung disease; DAH, Diffuse alveolar hemorrhage; RPGN, Rapidly progressive glomerulonephritis; ENT, ear, nose, throat; BVAS, Birmingham Vasculitis Activity Score; FFS, Five factor score; EUVAS, European Vasculitis Study Group. ESRD, end stage renal disease.

**Table 3:** A summary of the prognosis in each cluster.

Characteristics	Cluster 1 (N=33)	Cluster 2 (N=75)	Cluster 3 (N=45)	Cluster 4 (N=36)
Age	Elderly	Eldest	Middle-aged	Elderly
Organ involvements				
ILD	Moderate	Frequent	Less Frequent	Most frequent
DAH	Moderate	Less Frequent	Less Frequent	Less Frequent
RPGN	Frequent	Moderate	Moderate	Less Frequent
Frequently accompanied other organ involvements		ENT, Nervous system	Cutaneous, Mucous Membranes/eyes	
Laboratory findings				
Kidney function	The most severe	Slightly severe	Slightly severe	Normal
Systemic inflammation	Severe	The most severe	Severe	Severe
Disease severity				
BVAS	High	Highest	High	Lowest
The frequency of “FFS>3”	Highest	Moderate	Moderate	Lowest
The frequency of EUVAS-defined “Systemic+Severe”	High	Highest	High	Lowest
Complications (for 10 years)				
Overall mortality	Moderate	Highest	Lowest	Moderate
Respiratory infection mortality	Lowest	Highest	Low	Low
ESRD rate	Highest	Less	Less	Lowest
Relapse rate	Highest	Frequent	Frequent	Lowest

ILD, interstitial lung disease; DAH, Diffuse alveolar hemorrhage; RPGN, Rapidly progressive glomerulonephritis; ENT, ear, nose, throat; BVAS, Birmingham Vasculitis Activity Score; FFS, Five factor score; EUVAS, European Vasculitis Study Group. ESRD, end stage renal disease.

### Evaluation of prognosis between each cluster

**Figure 2:**
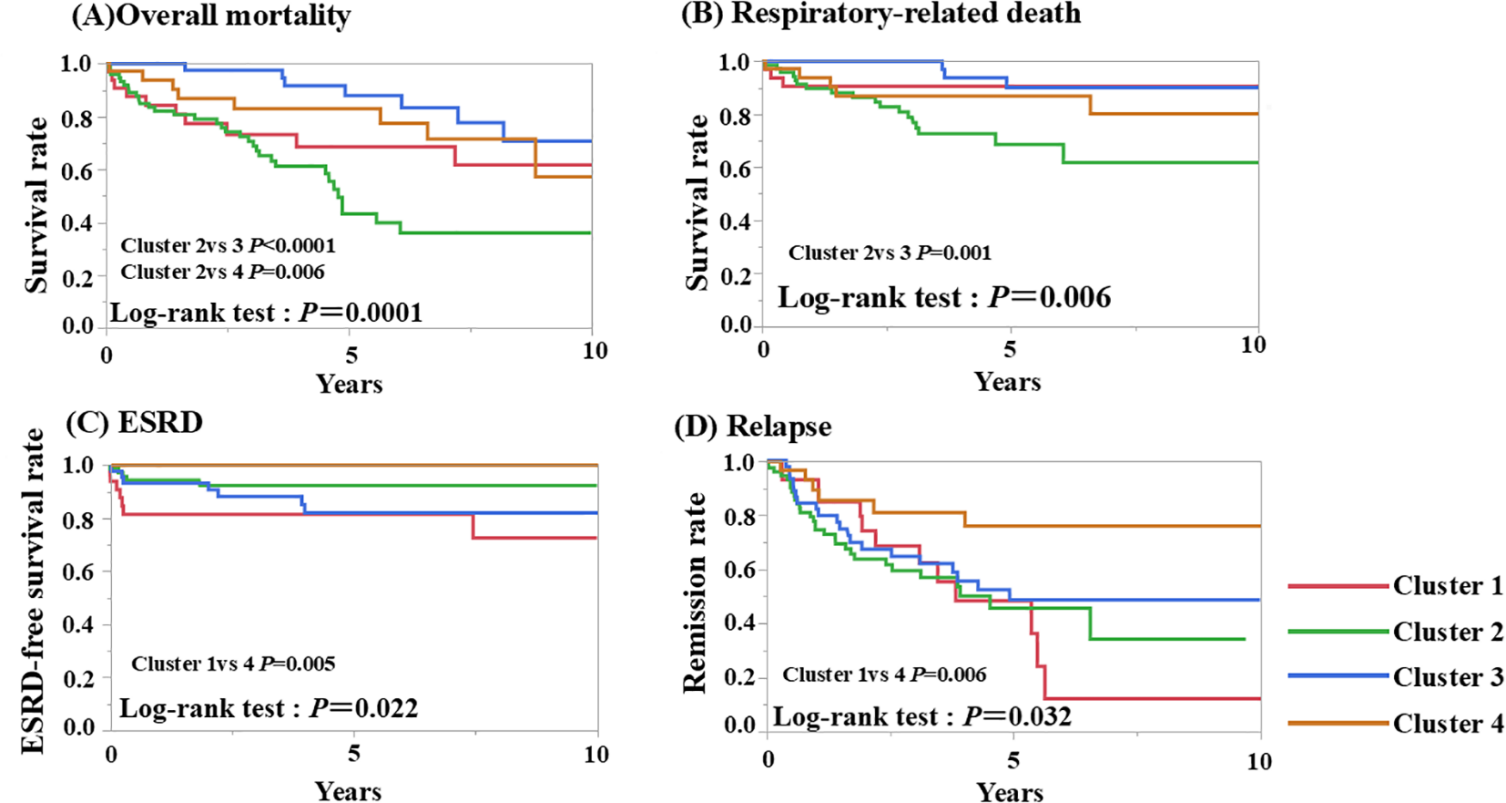
Kaplan-Meier curves for overall, respiratory-related death, ESRD, and relapse-free survival rate of four clusters. **(A)** Cumulative overall mortality -free survival rates, **(B)** respiratory-related death-free survival rates, **(C)** ESRD free survival rates, and **(D)** remission rates were analyzed using the Kaplan-Meier method. The log-rank test was used for intergroup comparison. ESRD, end-stage renal disease.

The overall survival, respiratory-related death-free survival, ESRD-free survival, and relapse rates among the four clusters were evaluated (**Tables 2**, **3**). Kaplan-Meier survival curves were plotted to estimate the probability of survival between the four clusters (**Figure 2**). 61 patients died after a mean follow-up of 4.4 years. Of these, 32 died due to respiratory-related deaths. Details of the cause of overall mortality are shown in **Supplementary Table 6**. As for infection-related deaths, 25 died due to respiratory-related infection and 7 died due to non-respiratory-related infection. The overall and respiratory-related survival rate in ten years, significantly differed across the groups (*P*=0.0002, 0.003, respectively). Among the four groups, patients in Cluster 2 had the worst overall survival and respiratory-related death-free survival rates in ten years (**Figures 2A, B**). The patients in Cluster 2 had a poorer prognosis than those in Cluster 3 in terms of overall and respiratory-related survival rate, with a statistically significant difference after Bonferroni correction (*P*<0.0001, *P*=0.0007, respectively).

In terms of ESRD rate and relapse rate, 19 patients developed ESRD, and 70 relapsed during follow-up. We found a significant difference in survival rate across the four groups in terms of ESRD rate and relapse rate (*P*=0.022, 0.032, respectively), and patients in Cluster 1 showed the worst ESRD-free survival and relapse rates. The patients in Cluster 1 had a poorer prognosis than those in Cluster 4 in terms of ESRD rate and relapse rate, with a statistically significant difference after Bonferroni correction (*P*=0.005, *P*=0.006, respectively) (**Figures 2C, D**). A summary of the prognosis in each cluster is shown in **Table 3**.

### Comparison of serial changes of clinical indicators between each cluster

Finally, we checked the serial changes of clinical indicators between each cluster. Laboratory findings and treatment context at the last observation for 121 patients were shown in **Supplementary Table 7**. The mean follow-up period after treatment was 4.3 years, and median prednisolone dose was 5 mg/day and 59.5% received immunosuppressants. The serum creatinine levels were significantly higher in Cluster 1-3 than Cluster 4. There were no significant differences in WBC, albumin, CRP, BVAS, PSL dose, or the ratio of immunosuppressants between them.

## Discussion

In this study, we conducted a categorical PCA-based cluster analysis using the clinical data of patients with MPA. Cluster analysis identified four subgroups: Cluster 1, renal involvement and diffuse alveolar hemorrhage (DAH) dominant group; Cluster 2, elderly onset systemic inflammation group; Cluster 3, younger-onset limited-organ disease group; and Cluster 4, ILD-predominant group without kidney involvement. There were significant differences in prognosis among the four groups. Cluster 1 showed the worst ESRD-free survival and relapse rates, whereas Cluster 2 showed the worst overall and respiratory-related death-free survival rates among the four groups.

MPA is often accompanied by pulmonary–renal syndrome (PRS), which is characterized by a combination of diffuse alveolar hemorrhage (DAH) and glomerulonephritis (29). Previous reports have shown that 90% of patients with MPA involve the kidney, and 8%–36% of patients with AAV also have DAH (1, 30, 31). In the present study, all patients in Cluster 1 involved the kidney, 36.4% of which had DAH. Therefore, nearly one-third of the cases in Cluster 1 met the PRS criteria. Previous reports have shown frequent relapses of AAV in patients with PRS (32, 33). Gallagher et al. reported that 25% of patients with PRS, including those with MPA, SLE, and GPA, were dialysis-dependent after 2 years of follow-up, and 4 out of these 14 patients relapsed 5 times during the follow-up (32). Kostianovsky et al. also showed that out of 80 patients with AAV and DAH, 76.3% were diagnosed with PRS, and 47 relapsed during follow-up (33). These reports support the results of the present study, which showed that the ESRD and relapse rates in Cluster 1 were the highest among the four clusters. This implies a potential need for more aggressive initial treatment therapy in Cluster 1 patients with MPA. In addition, prednisolone and immunosuppressive therapy should be carefully tapered during maintenance therapy to reduce the risk of relapse in these patients.

Patients with MPA in Cluster 2 were older and had high systemic inflammation/disease activity and a high frequency of systemic involvement, including general ENT, renal, and neuronal symptoms. Cluster 2 also showed worse overall survival and respiratory-related death-free survival rates than the other Cluster groups. In AAV, older age, systemic inflammation, and high disease activity are associated with poor prognosis (11, 34–36). Abe et al. and Koyama et al. previously reported that older age is associated with higher mortality in elderly patients with MPA and those with AAV-associated RPGN, respectively (34, 35). Watanabe et al. reported that patients with AAV and renal and high CRP levels (10 mg/dl) had worse survival rates (11). In addition, Itabashi et al. reported that the mortality rate of patients with BVAS ≧16 scores was significantly higher compared to those with BVAS< 16 scores in MPA (36). These findings support those of our study, showing that patients with MPA of older age, systemic inflammation, and high disease activity had a poor prognosis. Additionally, the patients in Cluster 2 showed the worst respiratory-related death rate among the four clusters because they had a high frequency of ILD (61.3%), resulting in complications such as infectious pneumonia, ILD exacerbation, and DAH (17, 37, 38). Also, systemic inflammation in patients in Cluster 2 may influence the high-rate complications of severe respiratory infections (39).

In contrast, the patients in Cluster 3 are younger and had a lower frequency of pulmonary and kidney involvement among the four groups. In addition, the total and respiratory-related mortality rates were the lowest. Cluster 3 had a better prognosis because these patients had fewer cases of severe renal and pulmonary involvement (6).

Patients in Cluster 4 had the highest complication rate of ILD without kidney involvement and the lowest systemic disease activity. Zhao et al. previously reported that patients with MPA‐UIP were less likely to have proteinuria and/or hematuria, and the degree of proteinuria in patients with MPA‐UIP was milder compared to those with MPA‐non‐UIP (40). This study supports our finding because a majority of HRCT patterns had a pattern of UIP in the present study. ILD frequently accompanies MPA and is included in the 2022 ACR/EULAR criteria for the diagnosis of MPA (41). Our study is the first to reveal a unique subtype of MPA in patients in the ILD-predominant group. Several reports have shown that MPA with ILD has a poorer prognosis than those with MPA without ILD (37, 42). However, conventional biomarkers, such as CRP and MPO-ANCA titers, cannot be used as predictive biomarkers for the prognosis of MPA-ILD (43, 44). In addition, the BVAS cannot evaluate the severity of ILD, because ILD is not included in the BVAS criteria (15). In the present study, the respiratory-related death rate in Cluster 4 was the second highest among the four groups; therefore, further studies will be needed to establish clinical indicators for poor prognosis in this ILD-predominant group.

Two studies used cluster analysis to identify clinical phenotypes of AAV (10, 11). Mahr et al. proposed five groups based on renal, cardiovascular, and gastrointestinal symptoms, as well as PR-3 ANCA in 673 patients, which included 396 patients with GPA and 277 with MPA (10). Watanabe et al. identified four subgroups based on MPO-ANCA, renal symptoms, and CRP levels in 427 patients with AAV (11). The subgroups included 86 patients with GPA, 270 with MPA, and 71 with unclassifiable MPA. However, no previous studies have focused on the clinical phenotypes of MPA. The present study revealed four unique subgroups of patients with MPO-ANCA-positive MPAs with different outcomes. In addition to the clinical significance of renal symptoms and CRP level, our study showed that age and pulmonary symptoms, including DAH and ILD, affected the prognosis of patients with MPA. The reason why our results differ from those of previous studies may be that the onset of MPA is higher than that of GPA, and these pulmonary symptoms are more frequently accompanied by MPA than GPA in the Japanese population (20, 22).

Our study has several limitations. All patients in this cohort were Japanese and MPO-ANCA positivity and ILD complication rates of MPA were high in this study. Therefore, it remains unknown whether our findings are applicable to other ethnicities. Second, our data related to prognosis may also have been affected by indication bias because the contexts of remission induction therapy and maintenance therapy for MPA were determined at the physician’s discretion at each institution. Third, cardiac and abdominal manifestations were not selected in the BVAS as variables, because these are rare manifestations of MPA. This may have led to an underestimation of the significance of these symptoms. Fourth, the participating centers in the REVEAL cohort were all tertiary referral hospitals. Our study has a high proportion of patients with high disease activity of MPA, so it may lead to tertiary care bias. Despite these limitations, our study highlights a new subgroup based on the clinical characteristics of MPA. Our multi-center REVEAL study involved a large dataset of 211 patients with MPA, with a median follow-up of nearly four years after immunosuppressive therapy; therefore, it may be suitable for identifying new subtypes. Further research utilizing a prospective multicenter model is required to determine treatment strategies based on these subgroups.

## Conclusions

Our study identified four unique subgroups with different MPA outcomes. Individualized treatments for each subgroup may be required to improve the prognosis of MPA.

## Data Availability

The original contributions presented in the study are included in the article/**Supplementary Material**. Further inquiries can be directed to the corresponding author.

## Supplementary material

Supplementary Figure 1 Scree plot for principal components analysis. X-axis shows the number of principal components. Y-axis shows the eigenvalues.

Supplementary Figure 2 Scree plot for cluster analysis. The scree plot has a point for each cluster join.
