# Supplementary figures and images for: Identification of novel clinical subtypes in patients with microscopic polyangiitis using cluster analysis: multicenter REVEAL cohort study
Source: Front Immunol. 2025 Jan 20;15:1450153. doi: 10.3389/fimmu.2024.1450153 (PMC11788177; doi:10.3389/fimmu.2024.1450153)

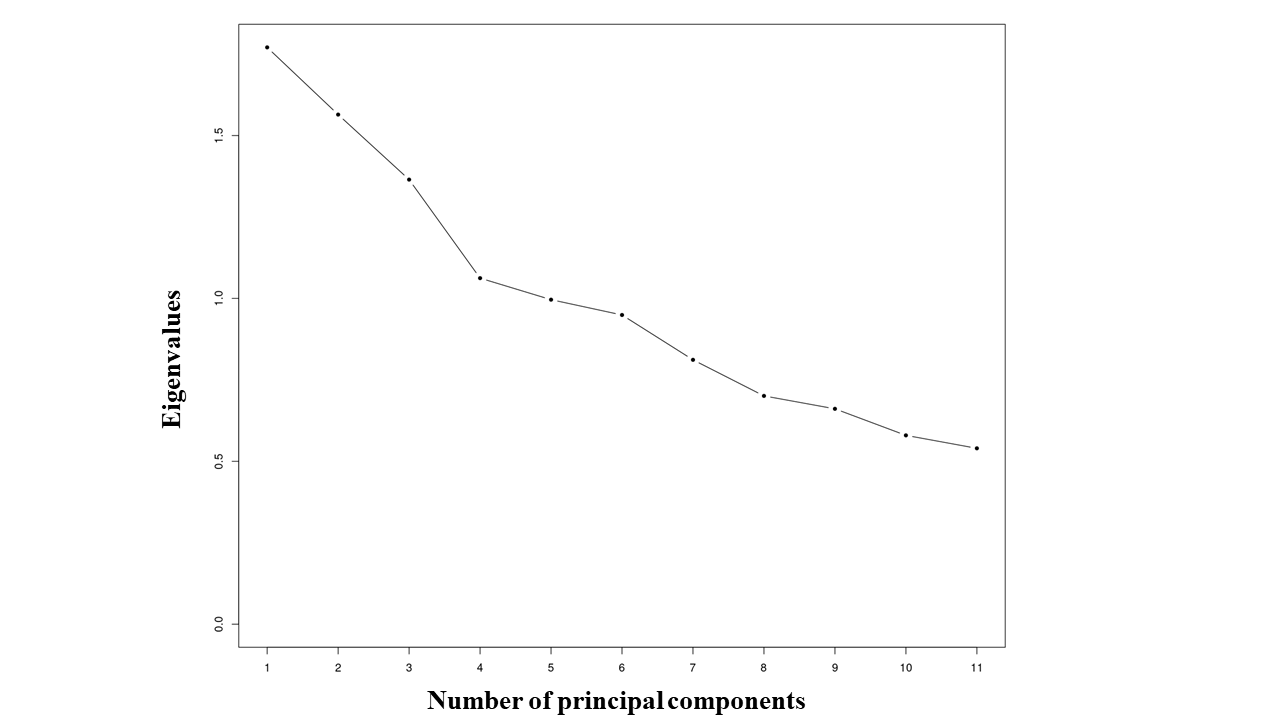

Supplement: Supplementary Figure 1 — Scree plot for principal components analysis. X-axis shows the number of principal components. Y-axis shows the eigenvalues. [file Image1.tif]

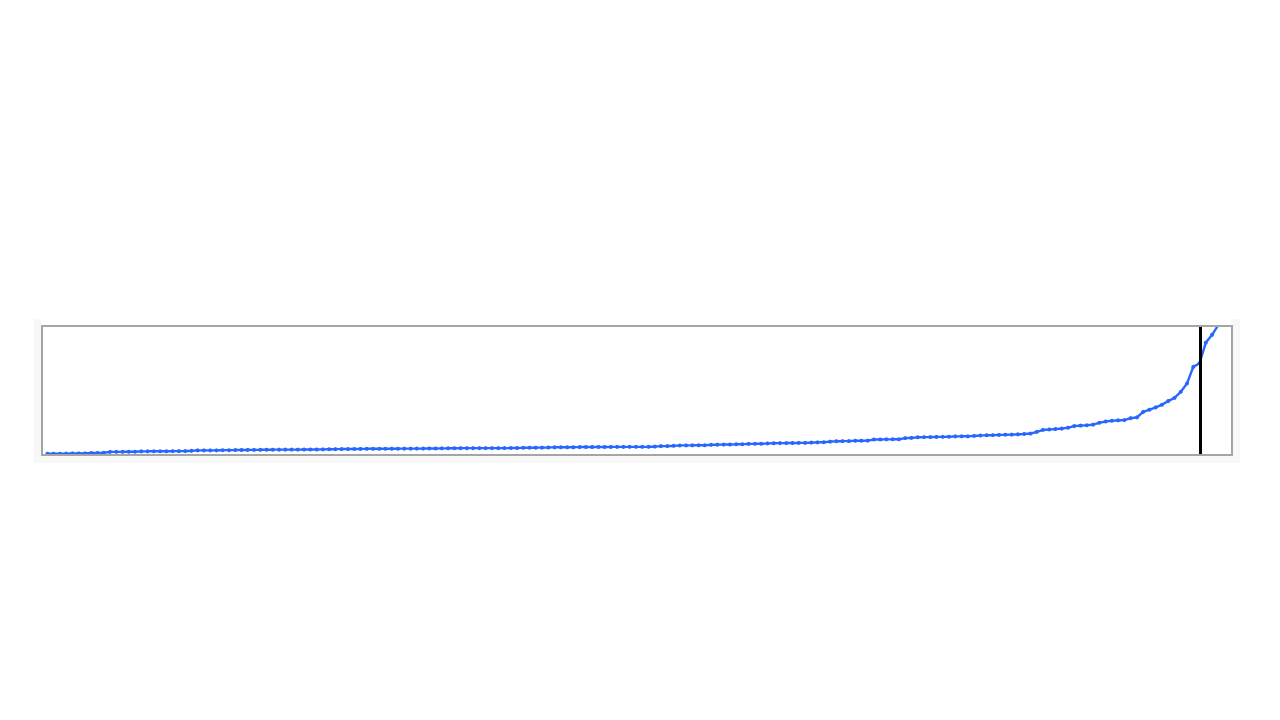

Supplement: Supplementary Figure 2 — Scree plot for cluster analysis. The scree plot has a point for each cluster join. [file Image2.tif]
