# Supplementary material for: Identification of novel clinical subtypes in patients with microscopic polyangiitis using cluster analysis: multicenter REVEAL cohort study
Source: Front Immunol. 2025 Jan 20;15:1450153. doi: 10.3389/fimmu.2024.1450153 (PMC11788177; doi:10.3389/fimmu.2024.1450153)
Supplement: Supplementary file 3 [file Table1.docx]

**Supplementary Table 1. Comparison of patients diagnosed with MPA between 2005 and 2012 and those diagnosed between 2013 and 2021in the REVEAL Study**

| Characteristics | 2005 to 2012  (N＝51) | 2013 to 2021  (N＝138) | *P* value |
| --- | --- | --- | --- |
| Age，years | 70(64-75) | 75(70-79) | 0.0021** |
| Female, n (%) | 32(62.8) | 71(51.5) | 0.16 |
| ILD, n (%) | 27(52.9) | 79(57.3) | 0.6 |
| **Laboratory findings** | |  |  |
| Cr, mg/dL | 0.9(0.65-2.03) | 1.19(0.75-2.15) | 0.23 |
| CRP, mg/mL | 9.9(4.7-12.4) | 6.9(2.8-12.3) | 0.16 |
| Positive anti-MPO-ANCA, n (%) | 50(98.0) | 137(99.3) | 0.49 |
| Positive anti-PR3-ANCA, n (%) | 2(3.9) | 7(5.1) | 0.74 |
| MPO-ANCA titer, U/mL | 92.3(43.5-231.3) | 125(62.5-253.1) | 0.25 |
| **Systemic symptoms** |  |  |  |
| General, n (%) | 36(70.6) | 85(61.6) | 0.25 |
| Cutaneous, n (%) | 7(13.7) | 11(8.0) | 0.25 |
| Mucous Membranes / eyes, n (%) | 3(5.9) | 10(7.3) | 0.74 |
| ENT, n (%) | 4(7.8) | 25(18.1) | 0.066 |
| Chest, n (%) | 23(45.1) | 47(34.1) | 0.17 |
| Cardiovascular, n (%) | 1(2.0) | 4(2.9) | 0.71 |
| Abdominal, n (%) | 0(0) | 1(0.7) | 0.43 |
| Renal, n (%) | 35(68.6) | 100(72.5) | 0.61 |
| Nervous system, n (%) | 14(27.5) | 56(40.6) | 0.092 |
| **BVAS at onset** | 13(7-19) | 15(8-21) | 0.19 |
| **Five factor score 2009** |  |  |  |
| ≦1 | 12(23.5) | 25(18.1) | 0.42 |
| 2 | 32(62.8) | 72(52.2) | 0.19 |
| ≧3 | 7(13.7) | 41(29.7) | 0.019* |
| **EUVAS-defined disease severity** |  |  |  |
| Localized | 3(5.9) | 4(2.9) | 0.36 |
| Early systemic | 8(15.7) | 37(26.8) | 0.1 |
| Systemic | 27(52.9) | 78(56.5) | 0.66 |
| Severe | 13(25.5) | 19(13.8) | 0.065 |

The laboratory markers are presented as the median (interquartile range). The P-values were estimated using Kruskal Wallis test or chi-squared test. **P* < 0.05, ***P* < 0.01, ****P* < 0.001. MPA: microscopic polyangiitis; ILD: interstitial lung disease; Cr: creatinine; CRP: C-reactive protein; MPO-ANCA: myeloperoxidase-anti-neutrophil cytoplasmic autoantibody; PR3-ANCA: proteinase 3-anti-neutrophil cytoplasmic antibody; ENT: Ear, Nose and Throat; BVAS: Birmingham Vasculitis Activity Score; FFS: Five Factor score; EUVAS: European Vasculitis Study Group.
