# Supplementary material for: Identification of novel clinical subtypes in patients with microscopic polyangiitis using cluster analysis: multicenter REVEAL cohort study
Source: Front Immunol. 2025 Jan 20;15:1450153. doi: 10.3389/fimmu.2024.1450153 (PMC11788177; doi:10.3389/fimmu.2024.1450153)
Supplement: Supplementary file 4 [file Table2.docx]

**Supplementary Table 2. Baseline treatments for 189 patients with MPA in the REVEAL Study**

| **Initial treatment** | N=189 |
| --- | --- |
| PDN, mg/day | 45(35-55) |
| MPDN pulse, n (%) | 57(30.2) |
| **Immunosuppressants** |  |
| CY, n (%) | 75(39.7) |
| Total CY dose (g) | 1.7(0.8-3.1) |
| RTX, n (%) | 16(8.5) |
| IVIG, n (%) | 8(4.2) |
| AZA/MTX/MMF/TAC/MZB, n (%) | 101(53.4)/5(2.6)/3(1.6)/11(5.8)/6(3.2) |
| PEX, n (%) | 14(7.4) |

PDN: prednisolone; MPDN: methylprednisolone; CY: cyclophosphamide; RTX: rituximab; IVIG: intravenous immunoglobulin; AZA: azathioprine; MTX: methotrexate; MMF: mycophenolate mofetil; TAC: tacrolimus; MZB: mizoribine; PEX: plasma exchange.
