# Supplementary material for: Identification of novel clinical subtypes in patients with microscopic polyangiitis using cluster analysis: multicenter REVEAL cohort study
Source: Front Immunol. 2025 Jan 20;15:1450153. doi: 10.3389/fimmu.2024.1450153 (PMC11788177; doi:10.3389/fimmu.2024.1450153)
Supplement: Supplementary file 5 [file Table3.docx]

**Supplementary** **Table 3. Correlations of the 11 variables with the nine principal components derived from the categorical principal component analysis in the 189 MPA patients.**

|  | **Components** | | | | | | | | | | |
| --- | --- | --- | --- | --- | --- | --- | --- | --- | --- | --- | --- |
|  | 1 | 2 | 3 | 4 | 5 | 6 | 7 | 8 | 9 | 10 | 11 |
| **Renal** | **0.61** | -0.42 | -0.44 | -0.03 | -0.13 | -0.11 | 0.10 | -0.18 | -0.20 | 0.08 | -0.37 |
| **ILD** | **-0.58** | 0.07 | 0.12 | -0.14 | -0.40 | 0.21 | 0.32 | -0.46 | 0.03 | -0.29 | -0.16 |
| **Age** | **-0.56** | -0.13 | -0.52 | -0.26 | 0.21 | 0.06 | -0.36 | -0.24 | -0.05 | 0.30 | -0.10 |
| **CRP** | -0.02 | **-0.62** | 0.13 | 0.20 | 0.43 | 0.40 | 0.03 | -0.16 | 0.20 | 0.06 | 0.38 |
| **Nervous system** | -0.24 | **-0.62** | 0.30 | -0.40 | 0.13 | -0.42 | -0.08 | -0.02 | -0.07 | -0.32 | -0.06 |
| **Cutaneous** | 0.21 | -0.14 | **0.58** | 0.01 | -0.09 | 0.24 | -0.68 | -0.12 | -0.21 | -0.10 | -0.04 |
| **Chest** | -0.11 | 0.17 | -0.27 | **0.66** | 0.26 | 0.04 | -0.16 | 0.05 | -0.09 | -0.55 | -0.20 |
| **ENT** | 0.14 | -0.07 | -0.34 | -0.51 | -0.00 | 0.38 | -0.00 | 0.22 | -0.35 | -0.43 | 0.33 |
| **Mucous Membranses/eye** | 0.38 | 0.31 | 0.22 | -0.27 | 0.63 | 0.17 | 0.03 | -0.43 | 0.01 | -0.15 | -0.03 |
| **General** | -0.06 | -0.25 | 0.18 | -0.30 | 0.24 | 0.49 | 0.07 | 0.46 | 0.33 | 0.06 | -0.44 |
| **Serum Creatinine** | 0.43 | 0.01 | -0.45 | -0.15 | -0.25 | -0.24 | -0.34 | -0.14 | 0.55 | -0.18 | 0.07 |
| **Variance explained (%)** | 16.10 | 14.22 | 12.41 | 9.66 | 9.06 | 8.63 | 7.38 | 6.37 | 6.01 | 5.27 | 4.91 |
| **Cumulative Variance explained (%)** | 16.10 | 30.32 | 42.72 | 52.38 | 61.43 | 70.06 | 77.44 | 83.81 | 89.82 | 95.09 | 100.00 |

The first four components, with eigenvalues >1, were selected for the further cluster analysis. The variance of the four components were 16.1, 14.2, 12.4 and 9.7%. The variables with loadings of at least 0.55 in the absolute value of each component are shown in bold.
