# Supplementary material for: Identification of novel clinical subtypes in patients with microscopic polyangiitis using cluster analysis: multicenter REVEAL cohort study
Source: Front Immunol. 2025 Jan 20;15:1450153. doi: 10.3389/fimmu.2024.1450153 (PMC11788177; doi:10.3389/fimmu.2024.1450153)
Supplement: Supplementary file 6 [file Table4.docx]

**Supplementary Table 4. Comparison of synthetic variables between 4 Clusters**

|  | Cluster 1 (N=33) | Cluster 2 (N=75) | Cluster 3 (N=45) | Cluster 4 (N=36) | *P* value |
| --- | --- | --- | --- | --- | --- |
| **First SV** | 0.38(-0.01-0.91) | -0.15(-0.60-0.17) | 1.020(0.48-1.37) | -1.12(-1.58--0.85) | ＜0.0001*** |
| **Second SV** | -0.030(-0.46-0.35) | -0.80(-1.17--0.27) | 0.58(-0.17-1.16) | 0.64(0.40-1.45) | ＜0.0001*** |
| **Third SV** | -0.66(-1.07--0.24) | -0.42(-0.97--0.36) | 0.20(-0.17-1.05) | 0.68(0.28-1.22) | ＜0.0001*** |
| **Forth SV** | 1.21(0.92-1.50) | -0.42(-0.92-0.061) | 0.0066(-0.43-0.22) | 0.072(-0.55-0.86) | ＜0.0001*** |

The laboratory markers are presented as the median (interquartile range). The *P*-values were estimated using Kruskal Wallis test. **P* < 0.05, ***P* < 0.01, ****P* < 0.001. SV: synthetic variables
