# Supplementary material for: Identification of novel clinical subtypes in patients with microscopic polyangiitis using cluster analysis: multicenter REVEAL cohort study
Source: Front Immunol. 2025 Jan 20;15:1450153. doi: 10.3389/fimmu.2024.1450153 (PMC11788177; doi:10.3389/fimmu.2024.1450153)
Supplement: Supplementary file 7 [file Table5.docx]

**Supplementary Table 5.** **Comparison of treatments between clusters.**

| Characteristics | Cluster1 (N=33) | Cluster2 (N=75) |  | Cluster3 (N=45) | Cluster4 (N=36) | *P* value |
| --- | --- | --- | --- | --- | --- | --- |
| **Initial treatment** |  |  |  |  |  |  |
| PDN, mg/day | 50(40-60) | 45(35-55) |  | 40(30-55) | 40(30-50) | 0.07 |
| MPDN pulse, n (%) | 15(45.5) | 23(30.7) |  | 9(20.0) | 10(27.8) | 0.12 |
| **Immunosuppressants** |  |  |  |  |  |  |
| CY, n (%) | 17(51.5) | 34(45.3) |  | 17(37.8) | 7(19.4) | 0.03* |
| Total CY dose (g) | 1.4(0.6-3.2) | 1.7(0.6-2.6) |  | 1.5(1.2-4.0) | 2.3(0.7-3.1) | 0.58 |
| RTX, n (%) | 4(12.1) | 7(9.3) |  | 5(11.1) | 0(0) | 0.22 |
| IVIG, n (%) | 1(3.0) | 6(8.0) |  | 1(2.2) | 0(0) | 0.19 |
| PEX, n (%) | 7(21.2) | 6(8.0) |  | 0(0) | 1(2.8) | 0.003** |
| AZA, n (%) | 13(39.4) | 44(58.7) |  | 26(57.8) | 18(50.0) | 0.27 |
| MTX, n (%) | 1(3.0) | 0(0) |  | 4(8.9) | 0(0) | 0.02* |
| MMF, n (%) | 1(3.0) | 2(2.7) |  | 0(0) | 0(0) | 0.51 |
| TAC, n (%) | 2(6.1) | 4(5.3) |  | 3(6.7) | 2(5.6) | 0.99 |
| MZB, n (%) | 3(9.1) | 3(4.0) |  | 0(0) | 0(0) | 0.87 |

The laboratory markers are presented as the median (interquartile range). The *P*-values were estimated using Kruskal Wallis test or chi-squared test. **P* < 0.05, **P < 0.01. PDN: prednisolone; MPDN: methylprednisolone; CY: cyclophosphamide; RTX: rituximab; IVIG: intravenous immunoglobulin; PEX: plasma exchange; AZA: azathioprine; MTX: methotrexate; MMF: mycophenolate mofetil; TAC: tacrolimus; MZB: mizoribine.
