# Supplementary material for: Identification of novel clinical subtypes in patients with microscopic polyangiitis using cluster analysis: multicenter REVEAL cohort study
Source: Front Immunol. 2025 Jan 20;15:1450153. doi: 10.3389/fimmu.2024.1450153 (PMC11788177; doi:10.3389/fimmu.2024.1450153)
Supplement: Supplementary file 8 [file Table6.docx]

**Supplementary Table 6. Detailed causes of death in the REVEAL Study.**

| Characteristics | MPA (N= 189) |
| --- | --- |
| **All cause of death, n (%)** | 61 (32.2) |
| **Respiratory-related death, n (%)** | 32 (16.9) |
| Alveolar hemorrhage, n (%) | 4 (2.1) |
| Exacerbation of ILD, n (%) | 3 (1.6) |
| Infectious pneumonia, n (%) | 25 (13.2) |
| **non-respiratory-related death, n (%)** | 29 (15.3) |
| Vasculitis, n (%) | 1 (0.5) |
| Cardiovascular events, n (%) | 3 (1.6) |
| Cancers, n (%) | 2 (1.1) |
| Infection, n (%) | 7 (3.7) |
| GI bleeding, n (%) | 3 (1.6) |
| Cerebral hemorrhage, n (%) | 1 (0.5) |
| Cerebral infarction, n (%) | 1 (0.5) |
| Others, n (%) | 11 (5.8) |

ILD: interstitial lung disease; GI: gastrointestinal.
