# Supplementary material for: Identification of novel clinical subtypes in patients with microscopic polyangiitis using cluster analysis: multicenter REVEAL cohort study
Source: Front Immunol. 2025 Jan 20;15:1450153. doi: 10.3389/fimmu.2024.1450153 (PMC11788177; doi:10.3389/fimmu.2024.1450153)
Supplement: Supplementary file 9 [file Table7.docx]

| **Supplementary Table 7. Comparison of serial changes of clinical indicators between cluster 1 and 2 and　3 and 4 cases.** | | | | | |  |
| --- | --- | --- | --- | --- | --- | --- |
| Characteristics | Cluster 1 (N=20) | Cluster 2 (N=40) | Cluster 3 (N=36) | Cluster 4 (N=25) | *P* value | |
| **Laboratory findings** |  |  |  |  |  | |
| WBC, /mm^3^ | 8,250(6,720-9,700) | 8,160(5,790-10,140) | 8,240(4,720-9,490) | 8,850 (6,490-10,370) | 0.65 | |
| Alb, g/dl | 3.7(3.5-4.0) | 3.7(3.3-3.9) | 3.8(3.6-3.9) | 3.6(3.2-4.2) | 0.46 | |
| Cr, mg/dl | 1.22(0.97-1.98) | 0.98 (0.84-1.71) | 1.0(0.71-1.49) | 0.74(0.59-0.74) | 0.0003*** | |
| CRP, mg/dl | 0.2 (0.04-0.37） | 0.2（0.06-0.51） | 0.08(0-0.48) | 0.43(0.04-1.3) | 0.099 | |
| **Disease severity** |  |  |  |  |  | |
| BVAS | 0(0-0) | 0(0-0) | 0(0-0) | 0(0-0) | 0.57 | |
| **Treatment** |  |  |  |  |  | |
| PDN, mg/day | 5.5(3.3-7.9) | 5(5-8) | 5.5(3-9) | 5(5-8) | 0.84 | |
| Immunosuppressants, n (%) | 10(50.0) | 24(60.0) | 24(66.7) | 14(56.0) | 0.65 | |

The laboratory markers are presented as the median (interquartile range). The *P*-values were estimated using Kruskal Wallis test or chi-squared test. **P* < 0.05, ***P* < 0.01, ****P* < 0.001. WBC: white blood cell; Alb: albumin; Cr: creatinine; CRP: C-reactive protein; BVAS: Birmingham Vasculitis Activity Score; PDN: prednisolone.
